# Ground Radioactivity Distribution Reconstruction and Dose Rate Estimation Based on Spectrum Deconvolution

**DOI:** 10.3390/s23125628

**Published:** 2023-06-15

**Authors:** Hang Xu, Xianyun Ai, Ying Wang, Wenzhuo Chen, Zikun Li, Xian Guan, Xing Wei, Jianming Xie, Ye Chen

**Affiliations:** 1 State Key Laboratory of NBC Protection for Civilian, Beijing 102205, Chinavoldemort@yeah.net (W.C.); 2021020493@stu.cdut.edu.cn (Z.L.);; 2 Nuclear Technology Key Laboratory of Earth Science in Sichuan, Chengdu University of Technology, Chengdu 610059, China

**Keywords:** radioactive distribution, dose rate estimation, response matrix, deconvolution

## Abstract

Estimating the gamma dose rate at one meter above ground level and determining the distribution of radioactive pollution from aerial radiation monitoring data are the core technical issues of unmanned aerial vehicle nuclear radiation monitoring. In this paper, a reconstruction algorithm of the ground radioactivity distribution based on spectral deconvolution was proposed for the problem of regional surface source radioactivity distribution reconstruction and dose rate estimation. The algorithm estimates unknown radioactive nuclide types and their distributions using spectrum deconvolution and introduces energy windows to improve the accuracy of the deconvolution results, achieving accurate reconstruction of multiple continuous distribution radioactive nuclides and their distributions, as well as dose rate estimation of one meter above ground level. The feasibility and effectiveness of the method were verified through cases of single-nuclide (^137^Cs) and multi-nuclide (^137^Cs and ^60^Co) surface sources by modeling and solving them. The results showed that the cosine similarities between the estimated ground radioactivity distribution and dose rate distribution with the true value were 0.9950 and 0.9965, respectively, which could prove that the proposed reconstruction algorithm would effectively distinguish multiple radioactive nuclides and accurately restore their radioactivity distribution. Finally, the influences of statistical fluctuation levels and the number of energy windows on the deconvolution results were analyzed, showing that the lower the statistical fluctuation level and the more energy window divisions, the better the deconvolution results.

## 1. Introduction

When large-area radioactive contamination is caused by a nuclear and radiation terrorist attack or a major nuclear and radiation safety accident, quickly and accurately obtaining information on radioactive materials and environmental radiation levels is extremely important to the assessment of the accident status and development trend [1]. The traditional measurement methods can be divided into on-site sampling laboratory analysis measurement, on-site portable instrument measurement, vehicle-mounted radiation measurement, and aerial radiation measurement [2]. Among them, the aerial radiation measurement method plays an important role in the emergency monitoring of radioactive material releases due to its fast response speed and ability to cover large areas [3,4].

The one-meter AGL (above ground level) dose rate, representing the dose rate at a one-meter height above the ground, is an important indicator for the evaluation of the radiation level in the surrounding environment since the central point of the human body is about one meter. According to its level, necessary protective measures would be taken to protect the health of the workers or the public [5]. Since the measurement results of airborne platform radiation detectors are usually gamma energy spectra and count rates (cps), it is necessary to convert them into one-meter AGL dose rate to quickly assess the hazards of radiation pollution to the environment and personnel and formulate the next response measures [6]. Therefore, establishing the conversion relationship between airborne count values and ground dose rate is crucial. In recent years, there have been significant advances in unmanned aerial platforms and nuclear radiation detector technology internationally [7,8,9,10,11], but research and achievements in accurate modeling, data processing, and in-depth interpretation of information for unmanned aerial radiation monitoring systems are relatively limited. Wang et al. reconstruct radiation fields based on Bayesian inference and net function interpolation method inside a room [12,13]. The TV-H-1 inpainting equation and Cahn–Hilliard equation were applied to reconstruct radiation fields for radioactive wastes [14,15]. Sasaki M et al. established an analytical method to convert the count rates obtained by the aircraft detector to the one-meter AGL dose rate [16,17] and used artificial neural networks to visualize ambient dose rate distribution from airborne radiation monitoring [18]. Liu et al. introduced an algorithm that reconstructs the distribution of radioactive pollution by solving the detector response factor equations. The algorithm performed well when detecting heights of less than 50 m and could be used to reconstruct the degree of ground pollution range using measurement data obtained from the air [5]. Zhang improved the algorithm based on Liu’s work and verified its effectiveness through simulation [19]. Shi et al. explored the LASSO algorithm for radiation field reconstruction under sparse conditions, achieving good results, and analyzed the influence of the number of detectors on the positioning results [20]. However, when radioactive contamination occurs, there will be a continuous surface source distributed with a single nuclide or even multiple nuclides [21,22], and partitioning the ground radiation level accurately and reasonably based on aerial measurement data is significant for personnel protection.

In this paper, a reconstruction algorithm for the estimation of terrestrial radioactivity distribution was proposed based on gamma spectrum and deconvolution. By introducing the spectrum in the deconvolution process and conducting energy window division, accurate estimation of ground radioactive nuclide types and distributions could be achieved. Then, based on the dose rate response matrix obtained by Monte Carlo simulation, the estimation of the one-meter AGL dose rate could be realized. On the basis of the above research, the influences of statistical fluctuation levels and the number of energy windows on the deconvolution results were studied.

## 2. Methods

### 2.1. Establishment of Simulation Database

The radiation field of the ^137^Cs point source in three dimensions was simulated using SuperMC [23]. The simulations were performed using photon flux statistics cards with flux–dose conversion factors given by ICRP Report 74 [24]. Two billion source particles were sampled in each simulation to ensure that the statistical variance at each measurement point was less than 0.05.

**Figure 1:**
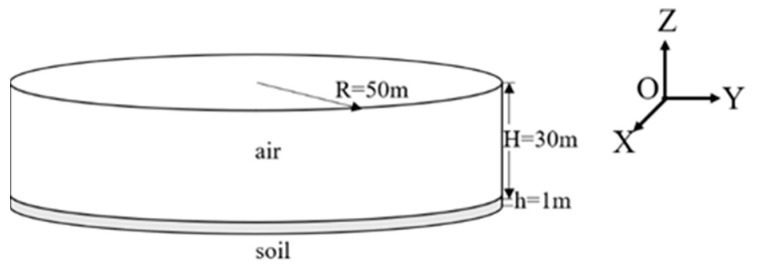
Spatial geometry model.

The spatial geometry model constructed was a cylinder with a radius of 50 m, where the soil was one meter in thickness with 30 m of air above, as shown in Figure 1. The interface between soil and air was set as the *Z* = 0 plane, with the geometric center of the plane as the origin, and the radioactive source (^137^Cs or ^60^Co) was located at the origin. Considering the influence of scattering, the statistical region was a square with a side length of 50 m centered on the point at a height of five-meter on the Z-axis where the radioactive source was located. Point detectors and three-inch NaI detectors were set at the grid nodes divided into 1 m × 1 m squares to obtain the air dose rate and detector spectrum at different positions.

### 2.2. Reconstruction Method of Single Nuclide Radioactivity Distribution

**Figure 2:**
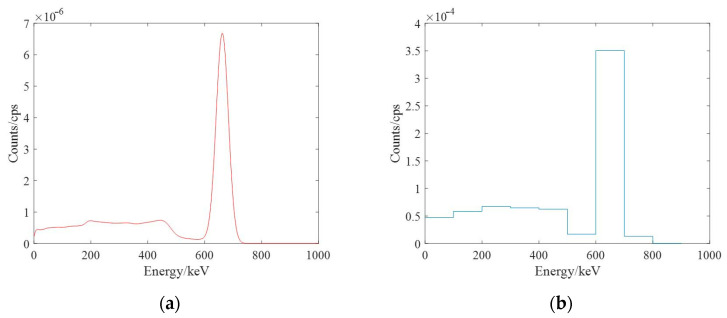
The γ spectrum of ^137^Cs: (**a**) original γ spectrum; (**b**) the spectrum after energy window division.

When using scintillator detectors for radioactivity measurements, the spectrum is a complex spectrum of multiple Gaussian-like peaks superimposed on the background due to the interactions of the incident γ-rays with the scintillator material, such as the photoelectric effect, Compton effect or electron pair effect, even if the incident γ-rays are monoenergetic. When a spectrometer is used for radiation field measurements, making use of the full information of the spectrum would help to determine the radionuclide species and distribution, and dividing the spectrum into several energy windows would help to find the characteristic peaks of the nuclide and then determine the nuclide species. Figure 2 shows the γ spectrum of ^137^Cs and the spectrum after being divided into 10 energy windows.

**Figure 3:**
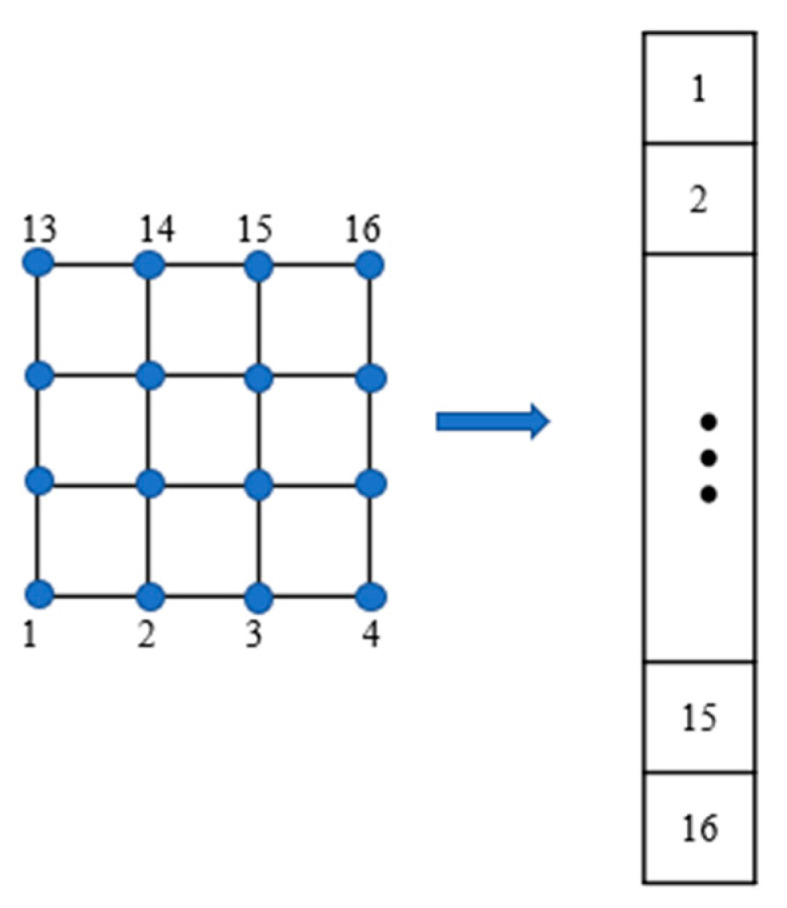
Schematic diagram of grid data conversion column vector.

Suppose the UAV (unmanned aerial vehicle) flies along the pre-set square grid path in the process of nuclear radiation monitoring and detects point by point on the grid nodes during the flight to get the gridded radiation monitoring data in the air, that is, the spectrum of each code. The spectrum was divided into k intervals, which would be with the total count data of the full spectrum k=1. The total number of measurement points was N. The ground was also divided into discrete grids with the total number as M. The value of each grid node was set as the radionuclide activity level at that location. The grid node data could be converted into column vectors as shown in Figure 3.

For the *n*th measurement point in the air, the relationship between the ground radiation intensity distribution and the spectrum could be expressed by Equation (1) when statistical fluctuations and noise were ignored.

(1) $$A_{n}\times x=y_{n},$$

where $A_{n}$ is the response matrix of the detector to radionuclides at the *n*th measurement point whose dimension is k×M, x is the source distribution vector at M×1, and $y_{n}$ is the spectrum of the n measurement point at k×1. The response matrix element $A_{n}(i,j)$ represents the contribution of the j ground grid to the i channel count of the spectrum $y_{n}$, and $A_{n}$ can be calculated using Monte Carlo simulation.

**Figure 4:**
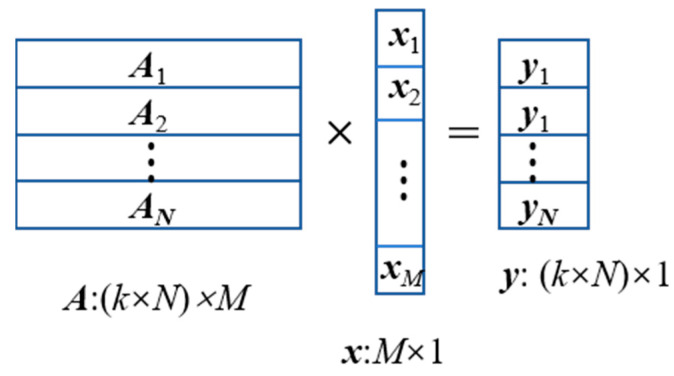
Detector response to ground radionuclide activity distribution.

For all measurement points, the relationship between radionuclide activity distribution and measurement results can be described as:

(2) A×x=y,

where A is the total response matrix composed of the response matrix of all measurement points, y is the column vector composed of the spectrum of all measurement points, and the detector response to the ground radionuclide activity distribution is shown in Figure 4.

**Figure 5:**
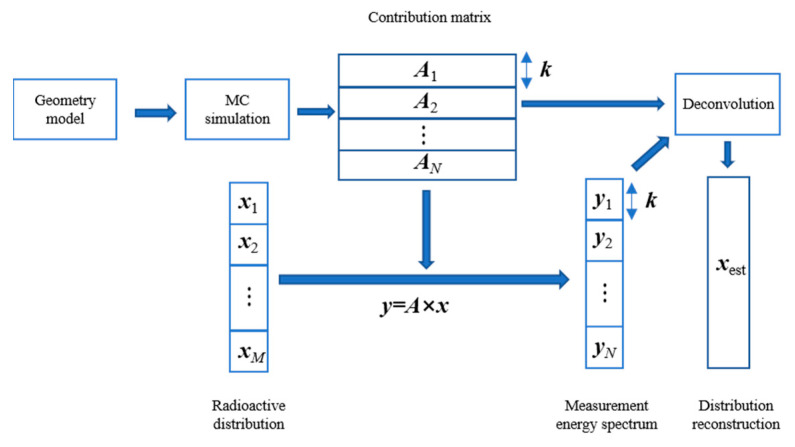
Diagram of radionuclide activity distribution reconstruction with γ spectrum.

Based on the above principle, when the response matrix of detectors to ground radionuclide activity and the spectrum of airborne detectors were obtained, the estimation of ground radionuclide species and distribution can be achieved by deconvolution. Figure 5 shows the reconstruction process of single radionuclide activity distribution based on the spectrum.

### 2.3. Reconstruction Method of Multi-Nuclide Radioactivity Distribution

**Figure 6:**
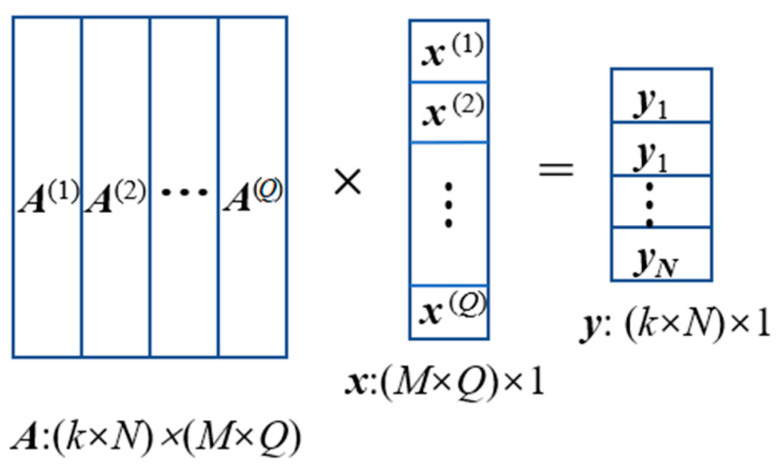
Multi-radionuclide detector response schematic.

When multiple radionuclides are present on the surface, each radionuclide contributes independently to the detector. Assuming a total of Q radionuclides, the response of the detector to ground radioactivity is shown in Figure 6. For all nuclides, there are:

(3) $$\sum_{q=1}^{Q}A^{\left(q\right)}x^{\left(q\right)}=y,$$

where $A^{\left(q\right)}$ is the response matrix for the *q*th nuclide, $x^{\left(q\right)}$ is the column vector of activity distribution of the *q*th nuclide on the ground grid, and y is the detector spectrum.

If the radionuclide species is known as a priori, with the activity distribution unknown, then $A^{\left(q\right)}$ only needs to build a response matrix for the known radionuclides, and the corresponding $x^{\left(q\right)}$ distribution can be obtained by spectrum deconvolution. If the radionuclide species is unknown, then a more comprehensive multi-nuclide spectrum response matrix needs to be built, the corresponding activity distribution can be obtained by deconvolution of the $x^{\left(q\right)}$ distribution, and the radionuclides present $x^{\left(q\right)}$ are not zero.

### 2.4. Dose Rate Estimation Method Based on Ground Radioactivity Distribution

If the radionuclide species and their distribution are known, since each radionuclide has its characteristic dose rate response matrix, the estimation of a one-meter AGL dose rate can be expressed using the model Equation (4):

(4) $$D=\sum_{q=1}^{Q}B^{\left(q\right)}x^{\left(q\right)},$$

where D is the distribution of one-meter AGL dose rate; $B^{\left(q\right)}$ is the M×M dimensional dose rate response matrix, which can be calculated using the Monte Carlo simulation; and $x^{\left(q\right)}$ is the activity distribution of the q radionuclide.

## 3. Results and Discussion

### 3.1. Single Radionuclide

**Figure 7:**
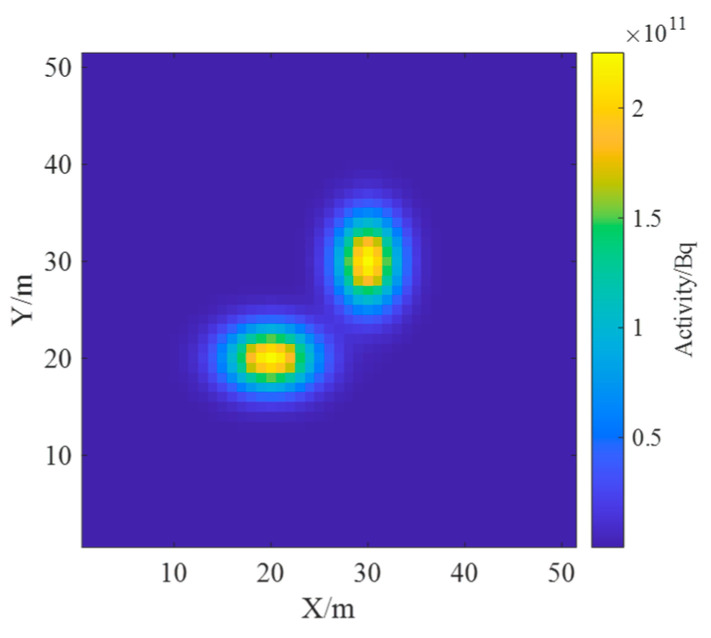
Ground radioactivity distribution of single radionuclide.

In order to verify the feasibility and effectiveness of the reconstruction algorithm of ground radioactivity distribution based on spectrum, an area surface source model was established, assuming that the radioactive contamination occurs only in the surface layer of the ground and the radionuclides are known to be ^137^Cs, so the spectrum was only concerned with the energy interval of 0~1 MeV. In this model, the ground was divided into a 50 × 50 size grid with a grid spacing of one meter. It was assumed that there were two ^137^Cs surface sources in the simulation area, both with an activity of 1 × 10^13^ Bq; the activity distribution obeys a two-dimensional Gaussian distribution; the discrete point sources were used to replace the area distribution surface sources, and all the point sources were located on the grid nodes. Surface source 1 was set with position parameter of $\left[\mu_{1x},\mu_{1y}\right]=[20,20]$ and covariance matrix of $\Sigma_{1}=\left[\begin{array}{cc} 10 & 0\\ 0 & 5 \end{array}\right]$. Surface source 2 was set with position parameter of $\left[\mu_{2x},\mu_{2y}\right]=[30,30]$ and covariance matrix of $\Sigma_{2}=\left[\begin{array}{cc} 5 & 0\\ 0 & 10 \end{array}\right]$. With these parameters, the real radionuclide distribution on the ground can be shown in Figure 7.

Airborne radiation detection simulations were performed in a plane five meters above the area with a grid spacing of two meters, and the number of measurement points was 26 × 26. Based on the ground radionuclide distribution, the detector spectrum of each node in the air was calculated in conjunction with the spatial radiation field simulation model in Section 2.1, and Poisson noise was added as the statistical fluctuations.

**Figure 8:**
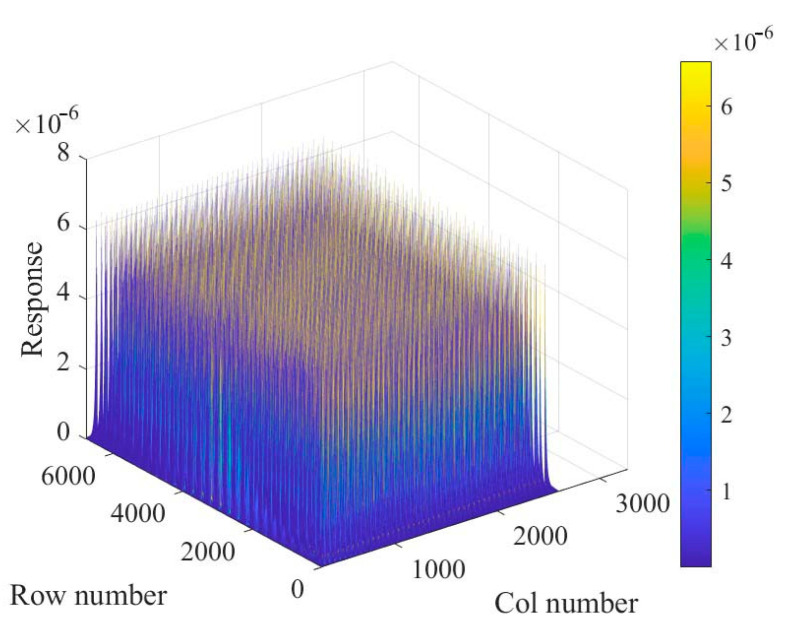
Single radionuclide response matrix based on spectrum.

**Figure 9:**
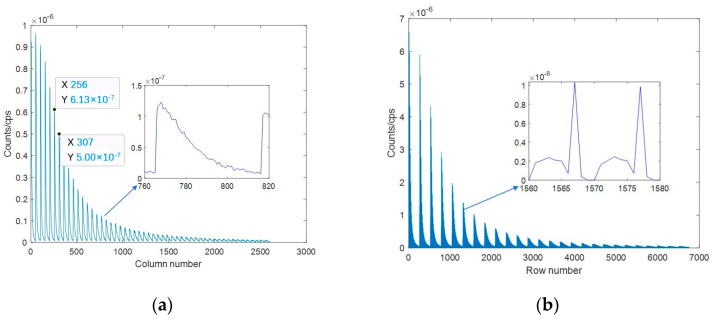
The response matrix based on spectrum. (**a**) Single row; (**b**) single column.

Each detector spectrum was uniformly divided into 10 channels, and the channel site energy interval is 100 keV, then the single nuclide response matrix based on the spectrum was constructed, as shown in Figure 8. Since the data dimension is too large, the first row and the first column are shown in Figure 9, respectively, from which it can be seen that the energy window counts would vary periodically with the peak of each node, and the counts would decrease gradually with the increase in the distance between the detectors and the nodes.

**Figure 10:**
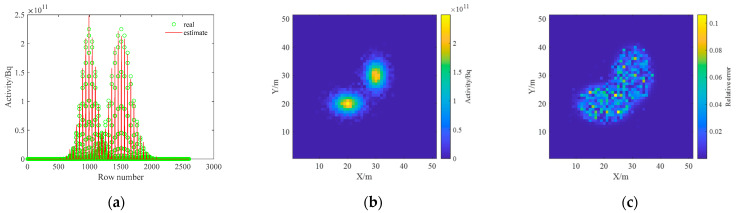
The reconstructed distribution of the ground activity of the single radionuclide. (**a**) One-dimensional; (**b**) two-dimensional; (**c**) relative error.

The inverse solution of the single radionuclide distribution using the non-negative least squares method was deconvoluted, and the ground radionuclide distribution was obtained as shown in Figure 10.

By comparing Figure 7 and Figure 10, it can be found that the reconstructed ground radionuclide distribution is similar to the actual ground radionuclide distribution and their maximum values are close. The cosine similarity and root mean square error between the inverse convolution estimation results and the real radioactive sources were calculated, and the results were 0.9955 and 2.9623 × 10^9^, respectively. The ground radionuclide distribution could be effectively restored using the reconstruction algorithm based on the spectrum.

**Figure 11:**
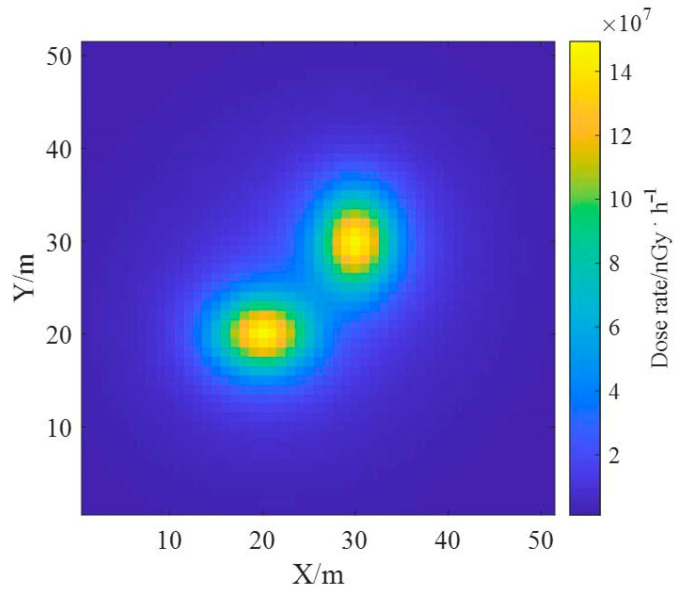
Estimated results of one-meter AGL dose rate.

An effective deconvolution calculation of the ground radionuclide distribution was performed by the reconstruction algorithm of the ground radionuclide distribution based on the spectrum, and the results were similar to the real distribution. Based on the results of radionuclide distribution deconvolution, the dose rate of a one-meter AGL was estimated, and the results are shown in Figure 11. It can be seen that the dose rate estimation results are smoother compared with the ground radionuclide distribution results, indicating that the influence of the inaccuracy of the ground radionuclide inversion results is attenuated after the dose rate estimation.

### 3.2. Multi-Radionuclide

**Figure 12:**
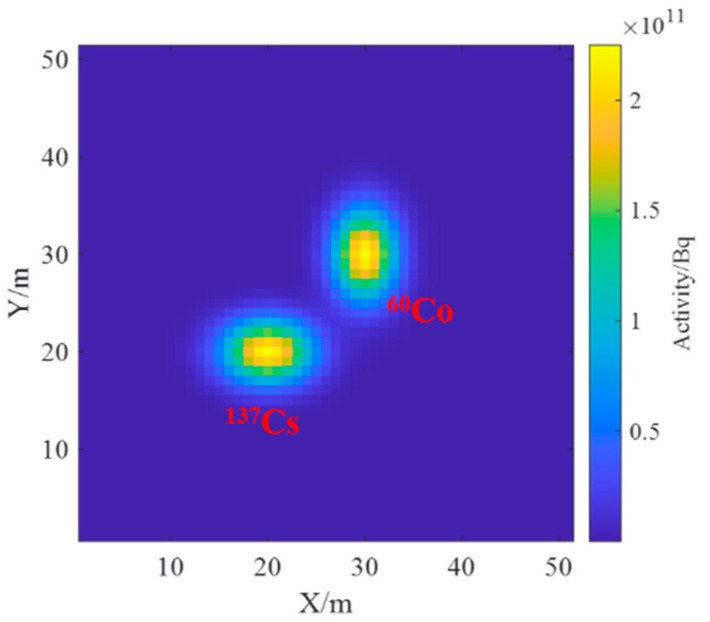
Ground radioactivity distribution of two radionuclides.

Based on the above model in Section 3.1, the radionuclides were set as ^60^Co and ^137^Cs with an activity of 1 × 10^13^ Bq. Surface source 1 was ^137^Cs with parameters of $\left[\mu_{1x},\mu_{1y}\right]=[20,20]$ and covariance matrix of $\Sigma_{1}=\left[\begin{array}{cc} 10 & 0\\ 0 & 5 \end{array}\right]$, and surface source 2 was ^60^Co with parameters of $\left[\mu_{2x},\mu_{2y}\right]=[30,30]$ and a covariance matrix of $\Sigma_{2}=\left[\begin{array}{cc} 5 & 0\\ 0 & 10 \end{array}\right]$. The real radionuclide distribution on the ground is shown in Figure 12. The spectrum information is only concerned with the energy interval of 0~1.5 MeV, and each detector spectrum is uniformly divided into 10 channels.

**Figure 13:**
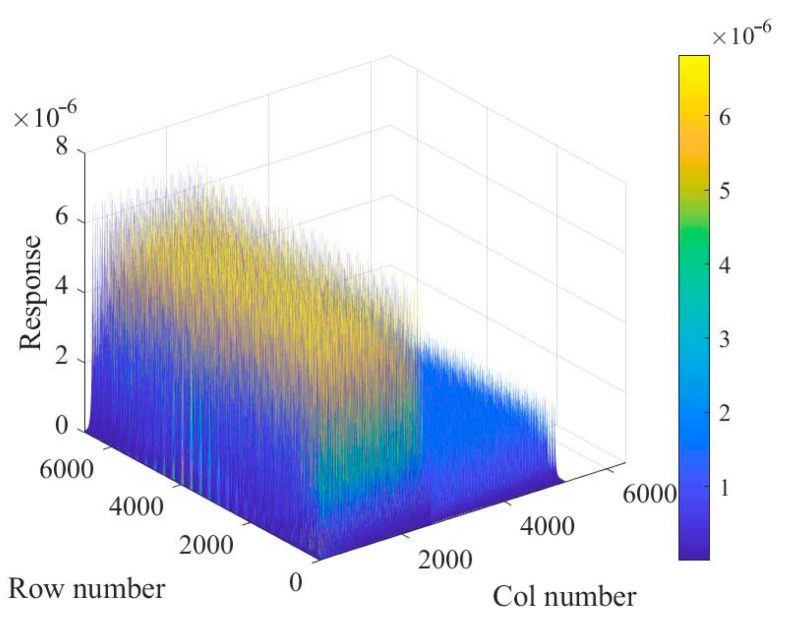
Response matrix of two radionuclides based on spectrum.

**Figure 14:**
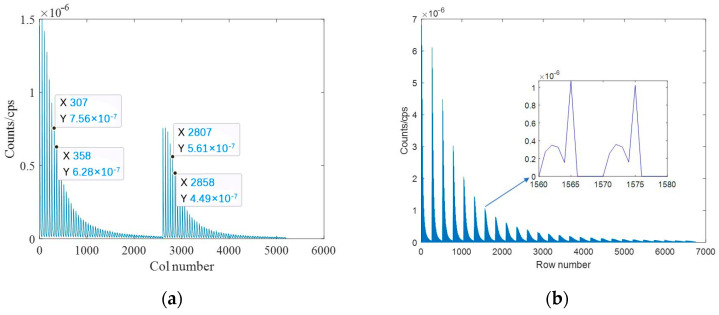
Response matrix of two radionuclides. (**a**) Single row; (**b**) single column.

The response matrix based on the spectrum was constructed as shown in Figure 13, from which it can be seen that the response matrix consists of two parts: the left part is the response matrix for ^137^Cs and the right part is the response matrix for ^60^Co. The first row and the first column are shown in Figure 14. It can be seen that the response matrix was divided into two parts, and the variation law of each part is the same as that of the single nuclide, but there is a difference in the response factor between the two nuclides.

**Figure 15:**
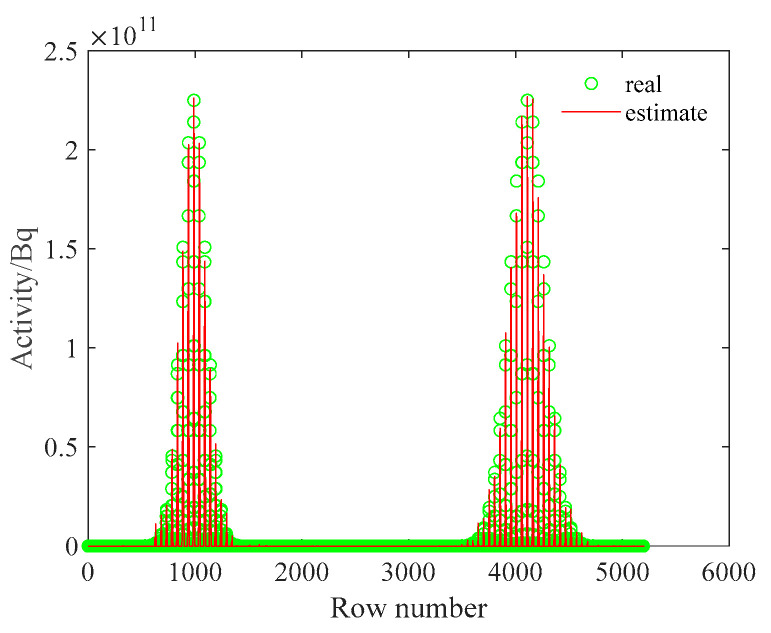
Diagram of the reconstructed one-dimensional distribution of the ground activity of two radionuclides.

**Figure 16:**
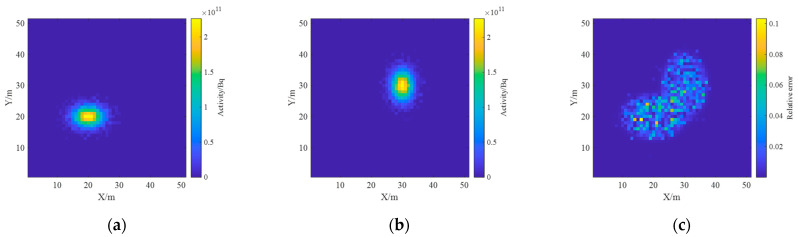
Diagram of the reconstructed two-dimensional distribution of the ground activity of the two radionuclides. (**a**) ^137^Cs; (**b**) ^60^Co; (**c**) relative error.

The inverse solution of the single nuclide activity distribution using the non-negative least squares method was deconvoluted to obtain the ground radionuclide activity distribution as shown in Figure 15. The conversion to a two-dimensional planar distribution is shown in Figure 16.

As can be seen in Figure 15, the distance between the two Gaussian peaks increases compared to that at the single-source radiation field; two different radionuclides can be identified and their activity distributions can be estimated separately. By comparing Figure 12 and Figure 16, it can be found that the distribution of two different radionuclides can be reconstructed separately using the spectrum-based ground radionuclide distribution reconstruction algorithm, the reconstructed ground radionuclide distribution is similar to the actual ground radionuclide distribution, and their maximum values are close. The cosine similarity and root mean square error between the inverse convolution estimation results and the real radionuclide sources were calculated, and the results were 0.9965 and 1.7410 × 10^9^, respectively, which proves that the ground radionuclide distribution reconstruction algorithm based on the spectrum can effectively distinguish between multiple radionuclides and restore their ground radionuclide distributions separately.

**Figure 17:**
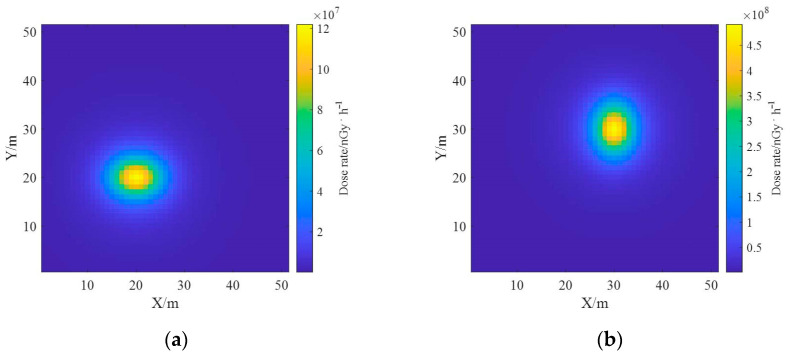
Estimation results of one-meter AGL dose rate for each radionuclide. (**a**) ^137^Cs; (**b**) ^60^Co.

**Figure 18:**
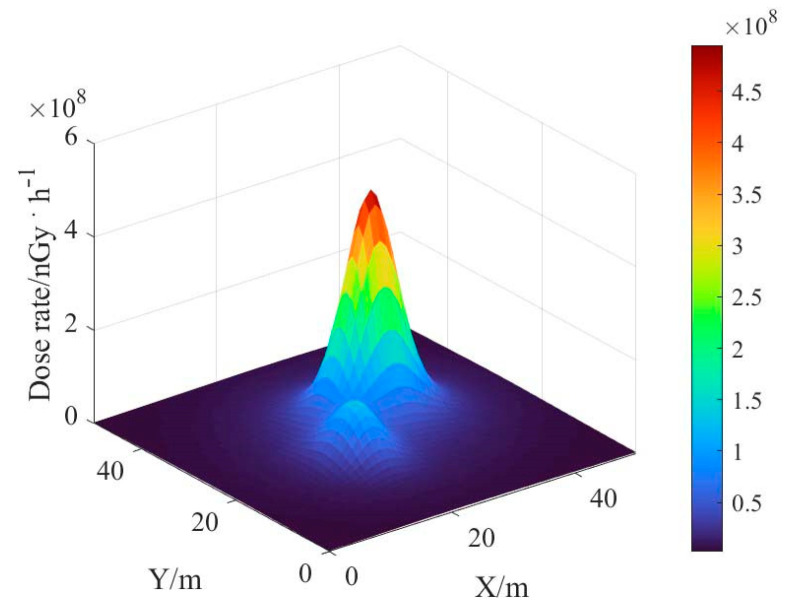
Estimation results of one-meter AGL dose rate for multi-radionuclide.

The ground radionuclide species have been distinguished based on the proposed reconstruction algorithm and the effective deconvolution calculations of their ground radioactivity distributions have been performed separately, and the results are similar to the real distributions. Based on the results of the deconvolution of the radioactivity distributions of the two nuclides, the one-meter AGL dose rates were estimated, and the dose rate distributions of the radionuclides ^137^Cs and ^60^Co obtained using the dose rate response matrix of the Monte Carlo simulation are presented in Figure 17. The total dose rate distribution in the region was obtained by superimposing the dose rate fields generated by both, as shown in Figure 18, from which it can be seen that the dose rate field generated by ^137^Cs with the same activity is smaller than that generated by ^60^Co.

### 3.3. Analysis of Influencing Factors

#### 3.3.1. Influence of Statistical Fluctuations

Since the γ spectrum counts obey Poisson distribution, the larger the count, the lower the statistical fluctuation level. Therefore, in this section, the number of spectrum channels k was kept constant, and only the total activity of radionuclides was adjusted to study the effect of the statistical fluctuation level on the deconvolution results. It was assumed that there were two radioactive surface source distributions with equal activity in the region, both of which are radionuclides ^137^Cs, and the spectrum was only concerned with the energy interval from 0 to 1 MeV.

**Figure 19:**
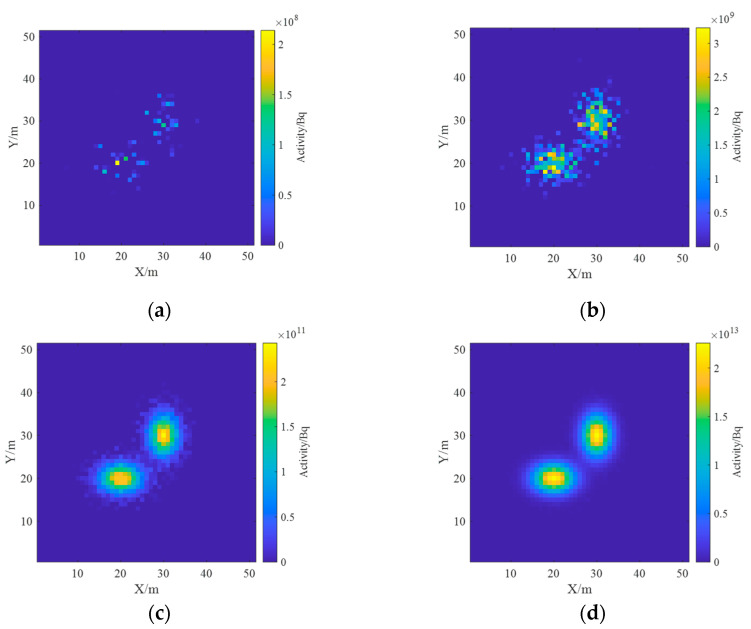
Reconstruction results of ground activity distribution at different total activity levels. (**a**) 10^9^ Bq; (**b**) 10^11^ Bq; (**c**) 10^13^ Bq; (**d**) 10^15^ Bq.

**Figure 20:**
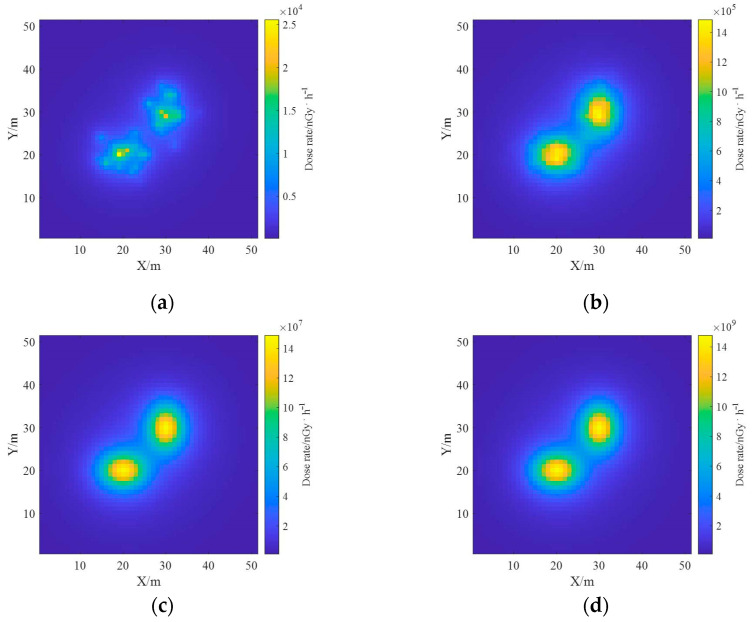
Dose rate estimation results at different total activity. (**a**) 10^9^ Bq; (**b**) 10^11^ Bq; (**c**) 10^13^ Bq; (**d**) 10^15^ Bq.

The reconstruction results of ground radioactivity distribution were obtained by setting the total activity of each surface source as 10^9^ Bq, 10^11^ Bq, 10^13^ Bq and 10^15^ Bq, respectively, and the mean RMSE (root mean square error) was calculated. Figure 19 shows the reconstruction results of radioactivity distribution deconvolution for four total activities of radionuclides, and it can be seen that as the total activity increases, the statistical fluctuation level is lower, the reconstruction results are closer to the actual situation, and the reconstruction effect is better. Figure 20 shows the estimated results of the one-meter AGL dose rate for four radionuclides with total activity. When the total activity is 10^9^ Bq, the dose rate can still obtain good results when the ground radioactivity distribution is estimated less accurately, which indicates that in the process of dose rate estimation, the influence of inaccuracy in nuclide activity distribution reconstruction could be effectively reduced due to the superposition effect.

**Figure 21:**
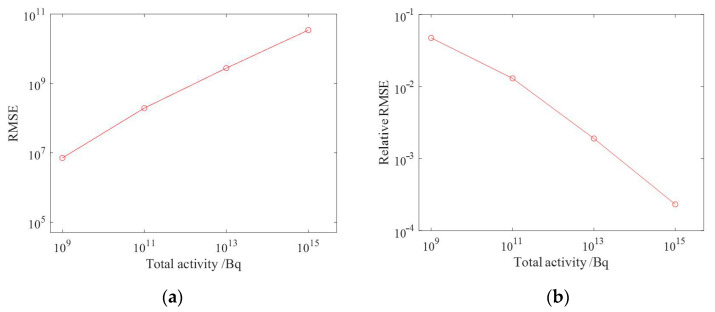
Results of the ground radioactivity distribution at different activity levels. (**a**) Mean RMSE; (**b**) mean relative RMSE.

**Figure 22:**
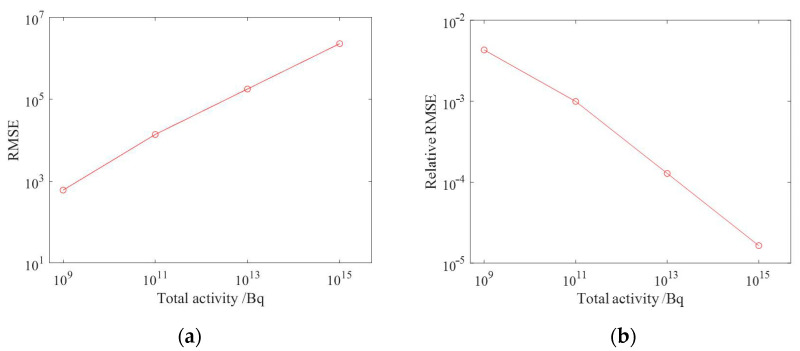
Results of dose rate estimation at different activity levels. (**a**) Mean RMSE; (**b**) Mean relative RMSE.

The average RMSE, average relative RMSE and cosine similarity were calculated, respectively, where the average relative RMSE was defined as the ratio of the RMSE value to the corresponding total activity, and the results of ground radioactivity distribution and dose rate estimation for different total activity conditions are presented in Table 1. Figure 21 shows the average RMSE and average relative RMSE of the ground radioactivity distribution of NNLS (non-negative least square) deconvolution with the total activity at different total activities. Figure 22 shows the average RMSE and average relative RMSE of dose rate estimation with the total activity at different levels of total activity. It can be seen that as the total activity of radionuclides increases, the absolute value of RMSE shows an increasing trend, while the relative RMSE shows a decreasing trend, which means the error of the deconvolution results would decrease as the statistical fluctuation level decreases, and the cosine similarity gradually converges to 1, indicating that the deconvolution results gradually approach the true results. Taking the relative RMSE of ground radioactivity distribution at a total radionuclide activity of 10^13^ Bq as the reference, the relative RMSE at a total activity of 10^9^ Bq is 24.9 times that of the reference value, while the relative RMSE at a total activity of 10^15^ Bq is only 0.12 times that of the reference value. It can be seen that the deconvolution results are strongly influenced by the statistical fluctuation level when the accurate system response matrix has been obtained. Therefore, in practical applications, parameters such as the minimum detectable activity of the detector should be taken into consideration to ensure that the measured counts can be sufficient to ensure the accuracy of the results.

#### 3.3.2. Influence of the Number of Energy Windows

Since dividing the detector spectrum into several energy windows can effectively obtain nuclide information, but the division of the energy windows leads to an increase in the response matrix dimension, the effect of the number of energy spectral channels k on the deconvolution effect was first investigated for the radiation field deconvolution problem at ^137^Cs, keeping the total activity constant.

**Figure 23:**
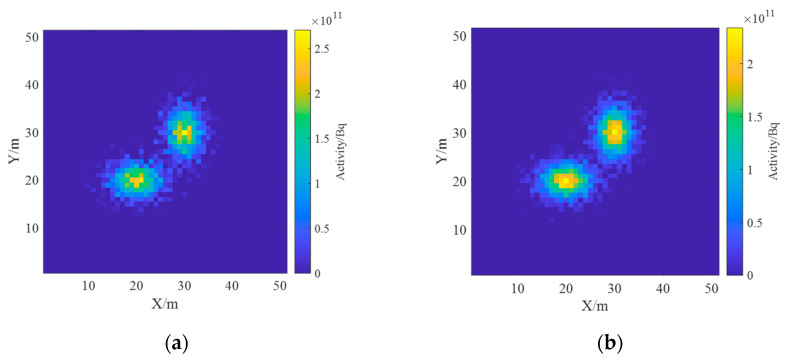

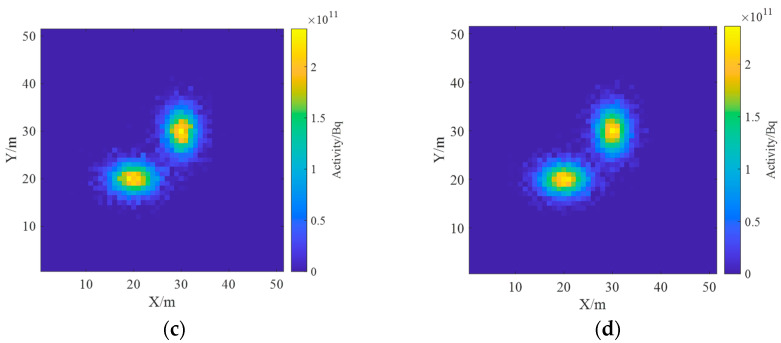
Comparison of the reconstructed ground activity distribution of single nuclide with different number of energy windows. (**a**) k = 1; (**b**) k = 2; (**c**) k = 5; (**d**) k = 10.

**Figure 24:**
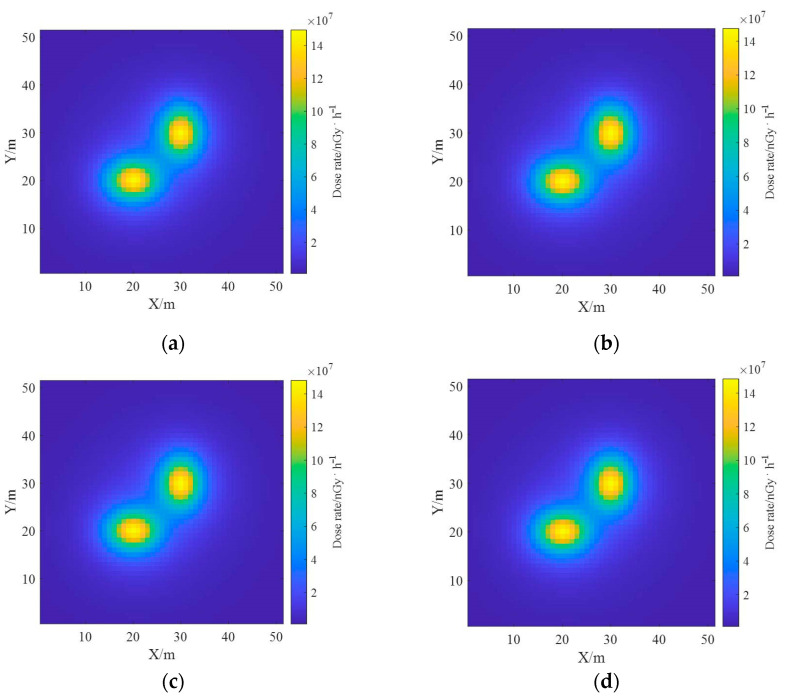
Comparison of single nuclide dose rate estimation results with different number of energy windows. (**a**) k = 1; (**b**) k = 2; (**c**) k = 5; (**d**) k = 10.

Since too many spectrum channel divisions will lead to an increase in computation time, this section only divides the spectrum into four cases of 1, 2, 5 and 10 channels respectively, with 1 channel being the full spectrum count, to obtain the deconvolution results, which are listed in Table 2. Figure 23 and Figure 24 show the reconstruction and dose rate estimation results of the ground radioactivity distribution deconvolution for the four cases, from which it can be seen that the increase in the spectrum channel number does not significantly improve the reconstruction results in the single nuclide case, but increases the computation time.

**Figure 25:**
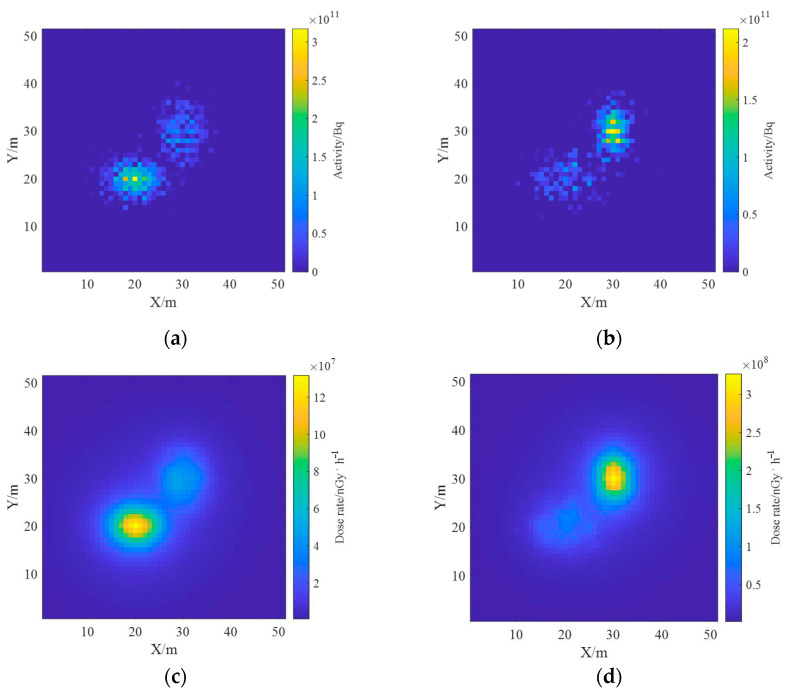
Deconvolution results of k=1 in the case of two radionuclides. (**a**) Activity distribution of ^137^Cs; (**b**) activity distribution of ^60^Co; (**c**) dose rate distribution of ^137^Cs; (**d**) dose rate distribution of ^60^Co.

**Figure 26:**
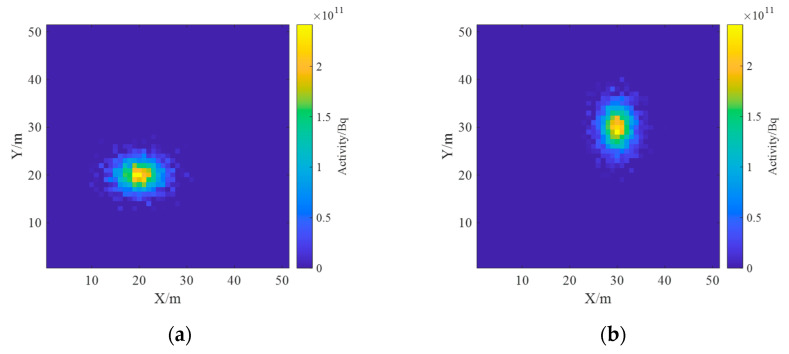

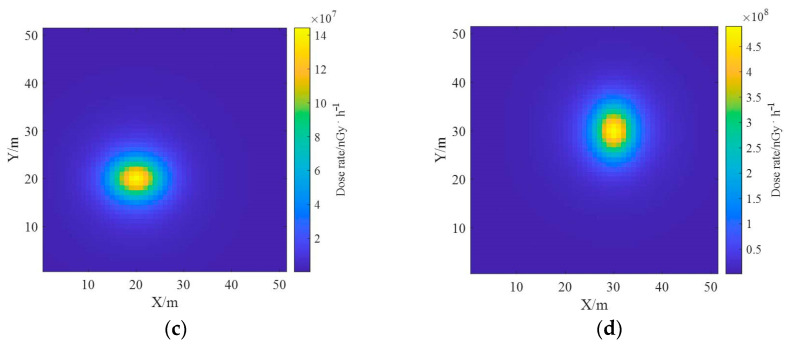
Deconvolution results of k=2 in the case of two radionuclides. (**a**) Activity distribution of ^137^Cs; (**b**) activity distribution of ^60^Co; (**c**) dose rate distribution of ^137^Cs; (**d**) dose rate distribution of ^60^Co.

Based on the physical model in 3.2.1, the cases of two radionuclides were explored to investigate the effect of the number of energy windows on the deconvolution results. The spectrum energy interval of interest here is from 0 to 1.5 MeV, and the estimated results for the two radionuclides are presented in Figure 25 and Figure 26 for the two cases with the number of energy windows of 1 and 2, with the results of the ground radioactivity distribution at the top and the results of the one-meter AGL dose rate estimation at the bottom.

It can be found that at k=1, the two radionuclides cannot be completely distinguished, and when k=2, two radionuclides, ^137^Cs and ^60^Co, can be distinguished. The reason is that when the spectrum is divided into two lanes, the ^137^Cs full-energy peak is located in lane 1, and the ^60^Co full-energy peak is located in lane 2, which do not interfere with each other, so these two radionuclides can be identified. However, the deconvolution result may not be satisfactory if the all-energy peaks of two nuclides are located in the same channel. Therefore, the energy window of the spectrum should be divided non-uniformly based on the priori information, so as to achieve an accurate estimation.

## 4. Conclusions

To address the problems of estimation of the ground radioactivity distribution and one-meter AGL dose rate, this paper proposes an algorithm for ground radioactivity distribution reconstruction based on a spectrum. By introducing the spectrum information in the deconvolution process and segmenting the spectrum according to the energy window, we achieved the estimation of ground radionuclide species and distribution based on the deconvolution method in the case that the radionuclide species and distribution are completely unknown, and then achieved the estimation of one-meter AGL dose rate based on the response matrix obtained from Monte Carlo simulation. The feasibility and validity of the algorithm were verified using two surface sources, single radionuclide and multi-radionuclides, and the results show that the cosine similarity between the estimated ground radionuclide distribution and the true value are 0.9950 and 0.9965; the method can effectively reconstruct the continuous distribution of multiple radionuclide and radionuclide distribution in the region, and the reconstructed results were in good agreement with the true value.

The effects of the statistical fluctuation level and the number of energy windows on the deconvolution results were investigated. The reconstruction results show that both of them have a great influence on the deconvolution results, and the lower the statistical fluctuation level and the more energy windows are divided, the better the deconvolution results are. Therefore, in practical applications, the UAV, due to its advantages of flexibility and mobility, can be used to measure the nuclear radiation area at low altitude, reduce the statistical fluctuation level, and combine the radionuclide a priori information as much as possible, and divide the energy windows appropriately to achieve accurate reconstruction of radionuclide distribution and dose rate estimation. In addition, energy resolution and background may also affect the reconstruction results to some extent, but these effects can be reduced by selecting detectors with good energy resolution and adopting some background subtraction methods.

## Figures and Tables

**Table 1:** Comparison of results at different activity levels.

Total Activity/Bq	Ground Radioactivity Distribution			Dose Rate Estimation		
	RMSE	Relative RMSE	Cosine Similarity	RMSE	Relative RMSE	Cosine Similarity
10^9^	7.12 × 10^6^	0.0474	0.3905	606.21	0.0043	0.9765
10^11^	1.96 × 10^8^	0.0130	0.8313	1.39 × 10^4^	9.95 × 10^−4^	0.9987
10^13^	2.79 × 10^9^	0.0019	0.9955	1.79 × 10^5^	1.28 × 10^−4^	1
10^15^	3.48 × 10^10^	2.31 × 10^−4^	0.9999	2.30 × 10^6^	1.65 × 10^−5^	1

**Table 2:** Comparison of calculation times with different number of energy windows.

k	Ground Radioactivity Distribution			Dose Rate Estimation			Deconvolution Time/s
	RMSE	Relative RMSE	Cosine Similarity	RMSE	Relative RMSE	Cosine Similarity	
1	6.69 × 10^9^	0.0045	0.9752	3.64 × 10^5^	2.61 × 10^−4^	0.9999	2.90
2	4.44 × 10^9^	0.0030	0.9888	2.67 × 10^5^	1.91 × 10^−4^	1	4.85
5	3.26 × 10^9^	0.0022	0.9939	2.02 × 10^5^	1.44 × 10^−4^	1	11.28
10	2.78 × 10^9^	0.0019	0.9955	1.79 × 10^5^	1.28 × 10^−4^	1	24.31

## Data Availability

The data that support the findings of this study are available from the corresponding author upon reasonable request.
